# Recent advances in managing and understanding enuresis

**DOI:** 10.12688/f1000research.11303.1

**Published:** 2017-10-24

**Authors:** Charlotte Van Herzeele, Johan Vande Walle, Karlien Dhondt, Kristian Vinter Juul

**Affiliations:** 1 Pediatrics, Department of Child Nephrology, Ghent University Hospital, Ghent, Belgium; 2 Pediatrics, Department of Child Neurology & Metabolism, Pediatric Sleep Center, Ghent University Hospital, Ghent, Belgium; 3 Ferring Pharmaceuticals, Copenhagen S, Denmark

**Keywords:** nocturnal enuresis, enuretic, enuresis

## Abstract

Enuresis, particularly in children during sleep, can be a debilitating condition, affecting the quality of life of the child and his or her family. The pathophysiology of nocturnal enuresis, though not clear, revolves around the inter-related mechanisms of overactive bladder, excessive nocturnal urine production, and sleep fragmentation. The first mechanism is more related to isolated nocturnal voiding, whereas the latter two are more related to nocturnal enuresis, in which circadian variations in arginine vasopressin hormone play a key role. A successful treatment would depend upon appropriately addressing the key factors precipitating nocturnal enuresis, necessitating an accurate diagnosis. Thus, advancements in diagnostic tools and treatment options play a key role in achieving overall success. This review summarizes recent advances in understanding the pathophysiology of nocturnal enuresis, diagnostic tools, and treatment options which can be explored in the future.

## Introduction

Enuresis is a cause of social, psychological, and emotional distress and carries a significant clinical burden ^1^. It commonly refers to nocturnal enuresis (NE), which is defined as involuntary voiding during sleep, at least once a month, when patients have been symptomatic for a minimum of three months ^2^. It may or may not be associated with other lower urinary tract symptoms (LUTSs) and thus can be classified as non-monosymptomatic NE (NMNE) or monosymptomatic NE (MNE), respectively. This leads to nights of disturbed sleep affecting a person’s quality of life (QoL), thus causing mood disturbance, daytime sleepiness, fatigue, and reduced work productivity ^3, 4^. Besides having implications for the patient, this condition can be stressful for the entire family ^5^.

Though primarily reported in children with a prevalence of about 20% at five years of age, NE tends to affect about 2% of adults ^1^. These data are often falsely interpreted as a high spontaneous resolution rate of NE, but in reality severe bed wetters (more than five wet nights per week) have only a 50% chance of achieving spontaneous resolution before adulthood, although for a mild to moderate condition, the prognosis may be more than 90%. The association of NE with sleep pattern is still debatable; some researchers associate enuresis with “deep sleep” related to a high arousal threshold ^6^ (although never evidence-based medicine-proven and largely subjective), whereas others correlate it with disturbed or “light sleep” with reduced feeling to wake up for voiding ^7^. In fact, a recent study has shown a correlation between the two theories, concluding that children with NE have greater awakenings caused by bedwetting, which in turn causes sleep fragmentation (cortical arousal) and hence a high arousal threshold. So they fail to respond to a full bladder and this ends up in a vicious cycle of additional bedwetting ^8^. Recently, newer data have attributed NE to periodic limb movement during sleep (PLMS) ^9^, which we discuss later in this review.

It is also worthwhile to discuss the role of comorbidities associated with NE, which is partially understood. Although NE seems to be associated with psychological and behavioral comorbidities such as attention deficit hyperactivity disorder (ADHD), autism spectrum disorder, anxiety, depression, and constipation ^2, 10^, a definitive relationship has not yet been established and needs to be further evaluated in order to rationalize the need to appropriately diagnose and manage the condition. Back in the nineties, Hägglöf *et al*. ^11^ showed that self-esteem is reduced because of enuresis and it improves/normalizes when the child becomes dry. Moreover, studies have shown that NE might lead to comorbid conditions such as ADHD symptoms and PLMS ^12^, besides causing sleep fragmentation ^8^. From these two findings, it can be interpreted that sleep deprivation (for example, reducing it by one hour a night) might lead to reduced daytime functioning with the nightly PLMS and that these symptoms may disappear when dryness is achieved and sleep restored ^13^. With these findings in view, it appears that enuresis and behavioral issues could have a cause-and-effect relationship, in which one might be stimulating the other, and it is difficult to say which stimulates what. Therefore, it is important to address the factors precipitating NE, which play a decisive role in treatment options. This review article therefore focuses on the recent advances in understanding the pathophysiology of NE, its diagnosis, and treatment options.

## Understanding the pathophysiology better

Over the years, our understanding of the pathophysiology of NE has advanced, making us realize that NE is far more complex than we understood three or four years ago. NE is generally associated with fragmented sleep, lower proportions of motionless sleep, and higher nighttime awakening ^8^, although a recent finding suggests that NE is associated with PLMS. The periodic limb movement disorder (PLMD) consists of involuntary movements of the limbs, seen as a flexion in the knee, hip, or ankle, particularly during the non-rapid eye movement phase of sleep, when the person is unaware of such movements. It impacts the patient’s QoL, as it perturbs the sleep at night and may cause daytime sleepiness. A pediatric study showed that children with NE had significantly higher PLMS, arousal, and awakening indices during sleep than children in the control group, who had a sleep disorder with reasons other than NE ^9^. The pathophysiology of PLMS is known to have its origin in dopaminergic neurotransmission ^14^. Dopamine depletion results in reduced inhibition of spinal cord-induced motor and sensory reflexes, which are reflected by the presence of PLMS and an increase in sympathetic outflow. In the pontine region, dopamine also has an important role in the micturition center and inhibits bladder contractions. Consequently, hypodopaminergic function results in reduced inhibition of spinal cord-induced muscle reflexes, which are reflected by the presence of PLMS and an increase of bladder contractions. Even though this is not completely understood, the co-occurrence of sleep fragmentation, PLMS, and NE, at least in some phenotypes of NE, is remarkable. This suggests that, in these patients, dopamine, sleep fragmentation, and thereby an increased sympathetic activity and higher cardiac rate and blood pressure (BP) during the night ^15^ might play an interactive and important role.

**Figure 1.**
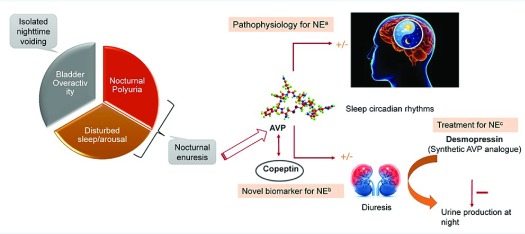
Interplay between arginine vasopressin (AVP) and nocturnal enuresis (NE): understanding it better. ( **a**) NE is caused by three related mechanisms: bladder overactivity (more related to isolated nighttime voiding), nocturnal polyuria (NP), and sleep disorders (more related to NE: AVP plays a key role by regulating diuresis and sleep circadian rhythms). ( **b**) Copeptin, an attractive alternative biomarker for AVP measurement, which is released in equimolar quantities as AVP and has greater plasma stability compared with AVP. ( **c**) Failure of the normal circadian rhythm of AVP in case of NE, resulting in large volumes of urine being produced at night. This led to the use of synthetic AVP analogue “desmopressin” as a treatment option.

Although the exact pathophysiology of NE remains partly understood, the data available to date suggest that NE is possibly caused by three related mechanisms—nocturnal polyuria (NP), bladder overactivity, and sleep disorder ^16^—which could be of urological, neurological, genetic, or psychological origin ^17^. Acetylcholine seems to play an important role in the pathophysiology of NE by increasing detrusor overactivity through its action on the M3 subtype of muscarinic acetylcholine receptors ^18^. The bladder overactivity is seemingly more related to isolated nighttime voiding, whereas NP and sleep disorders are seemingly more related to NE. The pituitary hormone arginine vasopressin (AVP) plays a key role in the pathophysiology of NP and sleep disorders by regulating diuresis and sleep circadian rhythms, respectively ^19^. As a result, a vicious cycle of sleep disruption and production of excessively large volumes of urine at night might lead to NE ( Figure 1). Children may have a large urine production during some nights and not during other nights and that is reflected in their AVP profile. So whether or not AVP is going to be measured, one has to realize that the outcome is different from wet nights to dry nights. AVP normally has a fairly consistent circadian rhythm, especially in pre-pubertal children, but may extend up to later in life for some children. The wet nights may be particularly associated with a lack of normal rise in plasma AVP, especially in the first part of the night ^20^. According to a theory that has recently surfaced, AVP circadian rhythms are not exclusively responsible for NP and in fact there are other intrinsic renal circadian systems that might be involved in the pathophysiology of NE ^21^.

Non-dipping BP during the night is another factor which leads to NE. Healthy individuals experience a nearly 10% decline in nocturnal arterial BP versus daytime arterial BP. In a study conducted in 45 children with MNE, dipping in systolic and diastolic BP based on a change of “<10%” was found to be significantly higher in patients than in healthy controls (n = 22; *P* <0.05) ^22^. Although the mechanism of these circadian changes in BP is not well understood, it might be a target of future NE treatment to enhance the BP dipping.

## Advancements in diagnosis

The initial diagnosis of NE is based on the subjective information provided by the patient or caregiver followed by self-filled diary data, which evaluate functional bladder volume and nocturnal diuresis ^23^. Most of the pediatric patients and their parents understand the importance of this crucial diagnostic tool, for which the validity is already established. However, these methods are sometimes inaccurate/biased and can lead to inappropriate diagnosis and treatment. In this scenario, it seems imperative to measure the plasma levels of AVP, not only for diagnosis but also for making treatment-related decisions and prognosis. However, measuring AVP accurately is practically challenging because of the unstable nature of the molecule and very short plasma half-life (about 20 minutes) ^24^. Also, more than 90% of the AVP is bound to platelets, precluding its accurate estimation. An attractive alternative in this regard could be a biomarker that is stable and easy to measure in laboratories even with basic infrastructure.

In this regard, copeptin, a cleavage product of peptide pre-pro-AVP during AVP synthesis, is being explored. The precursor peptide pre-pro-AVP is enzymatically cleaved into three components—vasopressin, copeptin, and neurophysin II—all released in equal proportions in the blood circulation. Recent advancements in biomarker research have delineated the use of copeptin as a surrogate marker for AVP measurements ( Figure 1) ^25^.

Copeptin (also known as CTproAVP), a 39-amino-acid glycopeptide, is the C-terminal part of the precursor peptide. Its level mirrors the levels of AVP in the body and changes with change in osmolarity. Compared with AVP, copeptin is much more stable in plasma ( *in vitro*) and during storage, does not require any pre-analytical processing and can be determined by using manual and automated assays ^25^. Moreover, copeptin concentration is independent of age ^26^, lacks consistent circadian rhythms ^27^ (unlike AVP, as discussed above), increases post-workout, and is higher in men than in women ^28^.

The role of copeptin as an important biomarker is already established and validated in various conditions where AVP plays a role. A recent study has shown its utility to differentiate central diabetes insipidus (DI) from nephrogenic DI. The former is caused by impaired AVP production/secretion from the posterior pituitary, and the latter is due to decreased sensitivity of the kidneys to AVP ^29^. Consequently, copeptin appears to be a promising new diagnostic biomarker to discriminate between different entities of polydipsia-polyuria condition, which consists of central DI, nephrogenic DI, and primary polydipsia. This differentiation is important, since inadequate treatment can lead to serious complications such as hyponatremia ^30^. Even in patients with autosomal dominant polycystic kidney disease, marked by high AVP levels, measuring copeptin levels can help to predict disease progression and severity ^31– 33^. In all of the above disease conditions, copeptin levels are seen to be directly correlated with AVP levels; however, the reverse is true in the case of chronic kidney disease (CKD). This is due to partial clearance of copeptin by the kidneys, hence increasing its plasma level in patients with CKD compared with those with preserved kidney function ^34, 35^. Copeptin also helps in the prognosis of CKD, as increasing copeptin levels predict a decline in estimated glomerular filtration rate ^36^.

Given the benefits of copeptin as a diagnostic and prognostic marker for AVP deficiency, it is now being explored for its diagnostic value in NE. However, until now, only one study has attempted to assess the relationship between copeptin and NE. This pediatric study (n = 88) showed that copeptin levels were significantly lower in patients with NE versus healthy controls. Within NE as well, copeptin levels were significantly lower in severe bed wetters (at least two nights per week) versus less-severe bed wetters (not more than one night per week). In the same study, however, AVP levels were not significantly different between any of the groups ^37^. This suggests that, compared with AVP, copeptin has a higher sensitivity in differentiating between the severity of NE and also better represents the low levels of AVP. This could be attributed to lower stability of AVP and also to the fact that a large fraction of AVP exists as platelet-bound as compared with copeptin.

So, if copeptin is going to have a role in the diagnosis of NE or even a role in the study of its pathophysiology, then a lot of studies lie ahead. Therefore, there is an emergent need to explore the utility of copeptin in establishing the diagnosis of NE due to NP and sleep disorders and in differentiating it from NE due to bladder overactivity. Once the diagnosis is established accurately, it will help physicians make better treatment-related decisions. Currently available treatments and recent advancements in this field are discussed in the following section.

## Newer therapeutic modalities

NE often remains un- or under-treated, primarily because of inappropriate or inadequate diagnosis despite the guidelines and recommendations in place. Treatment ideally should begin in children older than five years and in adults as soon as the condition is diagnosed.

An optimum treatment for NE would be the one that is based on a precise diagnosis and addresses the key underlying factor driving the disease pathophysiology. The recent consensus guidelines of the International Children’s Continence Society recommend education and motivational therapy to the child/parent/caregiver and behavioral advice to the child (diet/fluid restriction at night, voiding before going to bed) as the first-line treatment. In case of no respite, it is recommended to differentiate LUTS-associated NMNE from MNE, which is based on the voiding diary completed by the child/parent/caregiver, and once the diagnosis is ascertained, the treatment decision can be made. If the patient is diagnosed with MNE, the second-line treatment should preferably consist of a synthetic AVP analog—desmopressin (in case of NP only)—and an enuretic alarm (in case of reduced bladder capacity only). Moreover, a combination of desmopressin and alarm can be used in patients with both NP and reduced bladder capacity or when children using alarm therapy wake up more than once during the night ^23^. However, a minority of children with MNE are resistant to the standard treatment of either desmopressin or alarm therapy. Since in desmopressin-resistant patients, one of the mechanisms for enuresis seems to be excessive prostaglandin production at night, non-steroidal anti-inflammatory drugs (NSAIDs) such as indomethacin are suggested as a therapeutic alternative ^38^. Apart from NSAIDs, tricyclic antidepressants such as imipramine have come up as a useful alternate option, acting partly by reducing the clearance of solutes and partly by increasing urea and water reabsorption from the kidneys ^39^. Although imipramine proves to be cardiotoxic when overdosed, thus limiting its use in this condition, newer antidepressant options such as reboxetine have shown benefit. A recent placebo-controlled study in which enuretic children were given reboxetine 4 mg as monotherapy and as combination with desmopressin showed significant reduction in wet nights versus placebo ( *P* = 0.002) ^40^.

In case of NMNE, it is recommended to treat the comorbidities before initiating enuresis treatment, which could consist of a combination of desmopressin and anticholinergics (for example, oxybutynin). This combination can also be useful in MNE patients who fail to respond to alarm and desmopressin combination therapy ^41^. Thus, it is clear that owing to the complex pathophysiology that varies among patients, one treatment option may not suit them all.

Desmopressin has long been the treatment of choice for NE, and the following recent studies support this claim. In a prospective study involving MNE children, desmopressin has shown immediate and long-term effects that are as great as those of alarm therapy or a combination of alarm and desmopressin ^42^. Another prospective open-label study in a similar patient population given desmopressin has shown an improvement in sleep pattern post-therapy by a significant decrease in PLMS index ( *P* <0.001) at six months from baseline and prolonged first undisturbed sleep period (or time to first nocturnal void; *P* <0.001). This is also the first study to confirm the relationship among enuresis, neuropsychological dysfunction, and sleep disorders in an MNE population ^42^. Desmopressin also seems to be an effective treatment option for NE in patients undergoing orthotopic neobladder reconstruction after radical cystectomy, where it significantly reduced the mean number of nocturnal voids ( *P* = 0.015). Also, the number of patients with not more than one episode of NE per week increased from 19% to 39% after taking desmopressin. In 42% of patients, there was an increase of a minimum of one to two hours of sleep until the first nocturnal void, thus improving sleep quality and QoL ^43^.

Although desmopressin has been the standard treatment for MNE, there are still 20 to 60% of children who do not respond to the treatment and are desmopressin-resistant. These patients have a low bladder volume and low diuresis volume with high urinary osmolality overnight. In contrast, the desmopressin-responding patients have large bladder volume and high diuresis volume with low urinary osmolality overnight. Thus, a voiding diary can definitely help to individualize therapy ^44^. Desmopressin resistance in patients often coincides with the presence of comorbidities (constipation, attention problems, sleep disorders, and mental and motoric disability), thus making it essential to treat them first if possible. The pathophysiology for NE in these patients might involve renal circadian rhythms other than that of AVP ^21^. This has enhanced the need for alternative treatment options, particularly in the less well-defined MNE, where apparently a significant number of patients with NMNE are included.

Recently, transcutaneous electrical neural stimulation therapy (TENS), which is an established treatment option for hyperactive bladder and NMNE, has shown benefits in managing MNE as well. In the latter group versus controls, this therapy significantly reduced the rate of wet nights ( *P* = 0.02) and increased the rate of dry nights ( *P* = 0.004) regardless of age or gender ^45^. Long-term urodynamic studies have shown that there is no change in bladder activity, so it seems that the effect can be attributed to coping. Even though TENS has shown some benefits in patients with MNE, future studies need to be carried out in order to explore this treatment option.

## Biomarker for treatment effectiveness

Although desmopressin is the recommended treatment option for MNE, a biomarker to predict drug effectiveness is mandatory. Recently, urinary aquaporin 2, the major urine concentration factor, has come up as a biomarker of desmopressin treatment effectiveness during therapy. This pediatric study in which patients were treated with 120 or 240 μg desmopressin orally disintegrating tablets was divided into responders and non-responders. After eight weeks of treatment, a significant correlation was observed between day/night ratio of aquaporin 2 and percentage of wet nights. In the responder group, there was a significant difference in the change in aquaporin 2 day/night ratio from before treatment to complete remission ( *P* = 0.0004) ^46^.

## Conclusions

Nocturnal enuresis in children as well as adults can be detrimental for their overall development and well-being. Although the pathophysiology is unclear, NE is associated with a high arousal threshold and fragmented sleep, often associated with PLMD. Appropriate diagnosis for the reason precipitating NE is essential in order to provide the right treatment option. Copeptin seems to be a promising biomarker for AVP measurement, and more studies are needed in the future to establish the cause of NE. Desmopressin is the recommended treatment option for MNE associated with NP, but in a few patients who are treatment-resistant, newer options need to be investigated. In summary, this review article addresses new possible avenues and illustrates that there is so much to do in the future. The major achievement during the last few decades is that children are taken seriously and that it is realized that enuresis is not just enuresis but a lot of different entities.
